# Intracellular Binding of Terfenadine Competes with Its Access to Pancreatic ß-cell ATP-Sensitive K^+^ Channels and Human *ether-à-go-go*-Related Gene Channels

**DOI:** 10.1007/s00232-022-00252-y

**Published:** 2022-06-28

**Authors:** Bernd J. Zünkler, Maria Wos-Maganga, Stefanie Bohnet, Anne Kleinau, Detlef Manns, Shivani Chatterjee

**Affiliations:** 1 Federal Institute for Drugs and Medical Devices, Kurt-Georg-Kiesinger-Allee 3, 53175 Bonn, Germany; 2 Institute of Pharmacology, Toxicology and Clinical Pharmacy, Technische Universität Braunschweig, Mendelssohnstr. 1, 38106 Braunschweig, Germany

**Keywords:** hERG channel, K_ATP_ channel, Terfenadine, Intracellular binding

## Abstract

Most blockers of both hERG (human *ether-à-go-go*-related gene) channels and pancreatic ß-cell ATP-sensitive K^+^ (K_ATP_) channels access their binding sites from the cytoplasmic side of the plasma membrane. It is unknown whether binding to intracellular components competes with binding of these substances to K^+^ channels. The whole-cell configuration of the patch-clamp technique, a laser-scanning confocal microscope, and fluorescence correlation spectroscopy (FCS) were used to study hERG channels expressed in HEK (human embryonic kidney) 293 cells and K_ATP_ channels from the clonal insulinoma cell line RINm5F. When applied via the pipette solution in the whole-cell configuration, terfenadine blocked both hERG and K_ATP_ currents with much lower potency than after application via the bath solution, which was not due to P-glycoprotein-mediated efflux of terfenadine. Such a difference was not observed with dofetilide and tolbutamide. 37–68% of hERG/EGFP (enhanced green-fluorescent protein) fusion proteins expressed in HEK 293 cells were slowly diffusible as determined by laser-scanning microscopy in the whole-cell configuration and by FCS in intact cells. Bath application of a green-fluorescent sulphonylurea derivative (Bodipy-glibenclamide) induced a diffuse fluorescence in the cytosol of RINm5F cells under whole-cell patch-clamp conditions. These observations demonstrate the presence of intracellular binding sites for hERG and K_ATP_ channel blockers not dialyzable by the patch-pipette solution. Intracellular binding of terfenadine was not influenced by a mutated hERG (Y652A) channel. In conclusion, substances with high lipophilicity are not freely diffusible inside the cell but steep concentration gradients might exist within the cell and in the sub-membrane space.

**Graphical Abstract:**

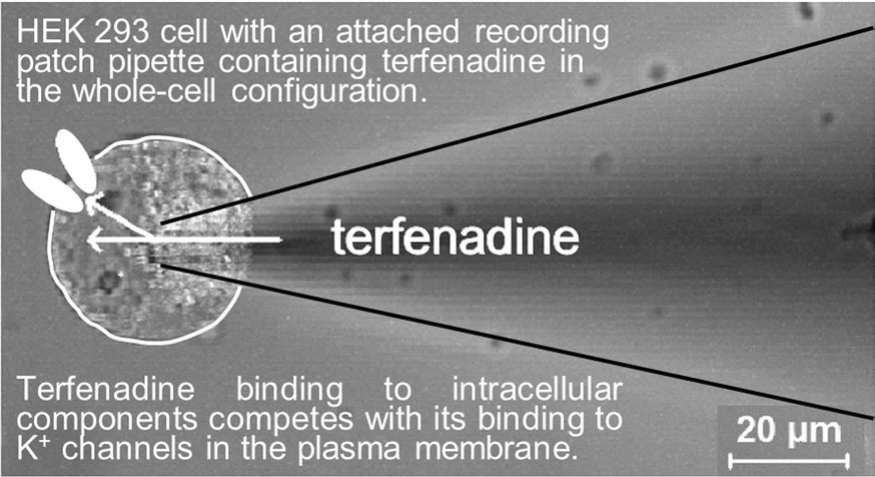

## Introduction

Block of hERG (human *ether-à-go-go*-related gene) currents by several non-cardiovascular drugs has the potential to induce QT interval prolongations in the electrocardiogram (ECG) and life-threatening Torsades de Pointes (TdP) cardiac arrhythmias (review in Keating and Sanguinetti (1996); for a compilation of torsadogenic drugs see www.crediblemeds.org). Most hERG channel blockers are basic compounds that cross the lipid bilayer of the plasma membrane as neutral molecules. In their protonated form, these substances gain access to their binding site located inside the central cavity after the channel has opened due to plasma membrane depolarization (Mitcheson et al. 2000; for recent reviews of the atomic-resolution structure of drug binding to the channel see Vandenberg et al. (2017) and Butler et al. (2020)). This model is supported by observations that permanently charged, membrane-impermeable analogs of cocaine (Zhang et al. 2001), verapamil (Zhang et al. 1999), ranolazine (Rajamani et al. 2008), and flecainide (Melgari et al. 2015) rapidly (within 10 min) inhibit hERG currents when applied intracellularly via the patch-pipette solution in the whole-cell configuration of the patch-clamp technique.

However, Melgari et al. (2015) and Du et al. (2011) observed that intracellular application of flecainide inhibits hERG currents to a lesser extent when compared to its extracellular application. A very slow development of hERG current block was observed after administration of a high (3 µM) concentration of astemizole via the pipette solution in whole-cell experiments (Fig. 3 of Taglialatela et al. 1998).

We used terfenadine, another non-sedating H_1_-antihistamine with a TdP-inducing potential like astemizole, to test whether its application via the pipette solution in whole-cell patch-clamp experiments has similar weak effects like astemizole on hERG currents. Terfenadine was one of the first substances for which the binding site on hERG channels has been identified: Mitcheson et al. (2000) demonstrated that the high-affinity binding site of terfenadine is located in the central cavity of the channel between the selectivity filter and the activation gate at Tyr652 and Phe656 in the S6 domain.

Furthermore, we tested the effects of terfenadine applied via the patch-pipette solution on K_ATP_ currents from the clonal insulinoma cell line RINm5F. Terfenadine inhibits these currents when applied via the bath solution, although at about 100-fold higher concentrations (with an IC_50_ value of 1.2 µM (Zünkler et al. 2000)) when compared to the concentrations inhibiting hERG currents. The pancreatic ß-cell ATP-sensitive K^+^ (K_ATP_) channel plays a central role in glucose-induced insulin secretion and is an octamer composed of four Kir6.2 and four SUR1 subunits (for a recent review of the function and of the atomic-resolution structure of the channel see Pipatpolkai et al. 2020). Terfenadine inhibits pancreatic ß-cell K_ATP_ currents via binding to the cytoplasmic side of the pore-forming Kir6.2 subunit (Zünkler et al. 2000), and the binding site for terfenadine on K_ATP_ channels of rabbit ventricular myocytes has been located close to the inner membrane surface (Nishio et al. 1998).

Therefore, terfenadine – like most other blockers of hERG and pancreatic ß-cell K_ATP_ channels – accesses its binding sites from the cytoplasmic side of the plasma membrane. We hypothesized that weak effects of intracellularly applied terfenadine on these ion channels can be explained by binding to intracellular components competing with its binding to K^+^ channels located in the plasma membrane. This hypothesis was tested using the whole-cell configuration of the patch-clamp technique, laser-scanning confocal microscopy, fluorescence correlation spectroscopy (FCS), and mutated hERG (Y652A) channels.

## Materials and Methods

### Transient Transfection of HEK 293 Cells with hERG/EGFP and Cell Culture

HEK (human embryonic kidney) 293 cells were transiently transfected with hERG/EGFP cDNA (coding for EGFP (enhanced green-fluorescent protein) tagged to the C-terminus of the hERG channel) as described previously (Claaßen et al. 2008).

HEK 293 cells stably expressing hERG channels were kindly provided by Prof. Dr. C. January (University of Wisconsin, USA) and cultured as described previously (Friemel and Zünkler 2010). Cells from the clonal insulinoma cell line RINm5F were cultured as described previously (Zünkler et al. 2004).

### Stable Expression of the Mutant hERG (Y652A) Channel in HEK 293 Cells

#### Construction of Plasmids

The basis of the plasmid constructions was pcDNA3-hERG which contains hERG (KCNH2, Kv11.1, GenBank accession U04270) under control of the immediate early cytomegalovirus (CMV) promoter. pcDNA3-hERG was generously provided by Prof. Dr. G. Robertson (University of Wisconsin, USA).

Tyrosine 652 of the hERG channel was mutated to alanine (Y652A) by using the QuikChange II XL site-directed Mutagenesis Kit (Stratagene). The employed primer pair was (5’-3’) CCTCATGTATGCTAGCATCGCCGGCAACGTGTCGGCCATCATCC and GGAGTACATACGATCGTAGCGGCCGTTGCACAGCCGGTAGTAGG. All constructs and the exchange of the coding triplets were verified by sequencing.

The mutation product pcDNA3-hERG (Y652A) was used to construct a green fluorescence protein (GFP) co-expression plasmid in the context of the plasmid pcDNA6.2/EGFP (Vivid colors of Invitrogen) as follows:

A PCR on the template of pcDNA3-hERG using the primer pair (5’-3’) hERG (GGTGAGCCACGTTCGTTCGAACACTGGGGAGCCCCCCTAACTGCCCGGGT) and CMV (GCAGTACATCAAGTGTATCATATGCCAAGTACGCCCCCTATTGACG) generated a fragment with hERG (Y652A). The boundaries of hERG (Y652A) were cut with Nde I at the 5’-end and with BstBI at the 3’-end. This NdeI, BstBI-cleaved fragment was ligated to the NdeI, BstBI-cleaved large vector fragment of plasmid pcDNA6.2/EGFP (Invitrogen) to generate the plasmid pcDNA6.2-hERG (Y652A), where hERG remains under the control of the CMV promoter.

#### Clone Generation and Selection of Stable Cell-Lines

Transient transfection of HEK 293 cells with plasmid pcDNA6.2-hERG (Y652A) was performed with the Lipofectamine 2000 reagent (Invitrogen) according to the manufacturer’s instructions. Transfected cells were cultivated in 6-well plates using 2 ml medium per well.

Selection of the stably transfected and fluorescent cells was performed using the Eppendorf micromanipulator PatchMan NP2.

### Electrophysiological Recording

K_ATP_ and hERG currents were recorded using the whole-cell patch-clamp configuration (Hamill et al. 1981) and an EPC-7 patch-clamp amplifier (Heka Elektronik, Lambrecht, Germany) as described previously (Friemel and Zünkler 2010; Zünkler et al. 2004). Stimulation protocols and data acquisition were carried out using a microcomputer equipped with D–A and A–D converters (Digidata 1440 Interface, Axon Instruments, Foster City, CA, USA) and pCLAMP 10 software (Axon Instruments). Current signals were filtered at 50 Hz for K_ATP_ channels and at 0.2 kHz for hERG channels, respectively, with the help of a Bessel filter (902, Frequency Devices, Haverhill, Massachusetts, USA), and the current sample frequency was 250 Hz for K_ATP_ channels and 1 kHz for hERG channels.

The following voltage-clamp protocol was used for the measurement of hERG currents: a holding potential of -80 mV, a voltage step to + 20 mV applied for 2 s to evoke hERG currents, followed by a repolarization step to -40 mV for 2 s to induce hERG tail currents (stimulation frequency of 0.1 Hz). hERG tail currents were leak corrected, and deactivating tail currents were fitted with two exponential functions and extrapolated to the beginning of the repolarization step in order to calculate the peak tail current amplitude. For the determination of the inhibitory effects of terfenadine and dofetilide on hERG currents after intracellular application, test substances were added to the pipette solution (D) (see Drugs and solutions section below). Peak tail currents were determined directly after breaking into the cells (time 0 min, control) and 10 min later.

In order to measure K_ATP_ currents from RINm5F cells using the whole-cell configuration, the membrane potential was held at -70 mV and hyper- and depolarizing voltage pulses of 10 mV amplitude and 200 ms duration were applied alternately every 2 s (Trube et al. 1986). For the determination of the inhibitory effects of terfenadine on K_ATP_ currents after extracellular application, 100 µM tolbutamide was applied in each experiment prior to the administration of terfenadine to estimate the contribution of K_ATP_ currents to the total currents evoked by the pulse protocol; only one concentration of terfenadine was tested in an experiment. For the determination of the inhibitory effects of terfenadine and tolbutamide on K_ATP_ currents after intracellular application, test substances were added to the pipette solution (C). Values for the maximum current density (in pA/pF) within 10–15 min after breaking into the cells were calculated by dividing the maximum current amplitudes to depolarizing pulses (10 mV) recorded from each cell by its membrane capacitance either in the absence of test substances (control) or in the presence of terfenadine or tolbutamide in the pipette solution.

At least 50% series resistance compensation was achieved in all experiments. Outward currents flowing from the pipette to the bath solution are indicated by upward deflections. Experiments were performed at room temperature (20–22 °C).

### Laser-Scanning Confocal Microscopy

The experimental setup consisted of both a patch-clamp apparatus and a laser-scanning confocal microscope, thus, allowing simultaneous laser-scanning confocal microscopy and electrophysiological experiments (Claaßen et al. 2008; Zünkler et al. 2004).

Laser-scanning confocal microscopy was used to measure the binding of 10 nM Bodipy-glibenclamide applied via the bath solution to RINm5F cells in the whole-cell configuration using pipette solution (C). The technique was essentially performed as described previously (Zünkler et al. 2004), but with slight modifications: an inverted Zeiss LSM-510 META laser-scanning confocal imaging system with an argon laser (excitation wavelength: 488 nm), a water immersion objective lens (C-Apochromat 40x/1.2) and a band-pass emission filter of 505 – 550 nm were used. The same laser-scanning confocal microscopy setup was used to localize hERG/EGFP fusion proteins expressed in HEK 293 cells.

Optical sections with a diameter of 1 µm across the axial (Z) axis were scanned through the middle of the cells at time intervals of 60 s. Intensity values for each cell were obtained by calculating the average brightness values of each optical section measured on an arbitrary gray scale from 0 (blackest) to 255 (whitest). In all experiments, confocal microscope settings (laser power, offset, and gain) were maintained constant. Laser-scanning confocal microscopy experiments were performed at room temperature (20–22 °C).

### Fluorescence Correlation Spectroscopy (FCS)

FCS measurements were performed on hERG/EGFP-expressing HEK 293 cells with a ConfoCor 2 fluorescence correlation spectrometer (Carl Zeiss Jena) using a C-Apochromat 40x/1.2 water immersion objective lens at room temperature (20–22 °C). The samples were excited using an argon laser (excitation wavelength: 488 nm), and fluorescence emission was detected using a band-pass emission filter of 530–600 nm. Spectra were recorded for 10 s, each single measurement was repeated 10 times, and the averaged results are shown in Fig. 6.

### Data analysis

The concentration–response relationships for the inhibition of peak tail current amplitudes by terfenadine (hERG and hERG (Y652A)) and dofetilide (hERG) were calculated as follows according to the logistic form of the Hill equation:

1 $$\frac{I}{{I_{c} }} = 1 - { }\frac{1}{{1 + { }10^{{n\left( {px - pK} \right)}} }}$$

For intracellular application, I/I_c_ is the ratio between the hERG or hERG (Y652A) peak tail current amplitude (I) at 10 min after breaking into the cells in the presence of different terfenadine or dofetilide concentrations applied via the pipette solution (D) and the control current amplitude (I_c_) obtained immediately after breaking into the cells. For extracellular application, I/I_c_ is the ratio between the hERG or hERG (Y652A) peak tail current amplitude (I) in the presence of different concentrations of terfenadine in the bath solution and the control current amplitude (I_c_) before application of terfenadine. x is the concentration of test substances, n represents the slope parameter (Hill coefficient), and K (= IC_50_) is the midpoint of the curve with px = -logx and pK = -logIC_50_.

The concentration–response relationship for the inhibition of K_ATP_ currents by terfenadine applied via the bath solution was calculated according to the following equation:

2 $$I = \left( {I_{c} - a} \right) \times \left( {1 - { }\frac{1}{{1 + { }10^{{n\left( {px - pK} \right)}} }}} \right) + a$$

where I_c_ is the current amplitude during the control period before application of terfenadine (control) and I is the current amplitude in the presence of different concentrations of terfenadine. The constant a in Eq. (2) was set to a value of 0.14 · I_c_ in order to describe the current component remaining in the presence of a high concentration (100 µM) of tolbutamide.

The concentration–response relationship for the inhibition of K_ATP_ currents by intracellularly applied tolbutamide was calculated according to Eq. (2), where I_c_ was set to a value of 18.6 pA/pF describing the mean current density in the absence of test substances in the pipette solution (control) and I designates the current density in the presence of different concentrations of tolbutamide in the pipette solution. Constant a was set to a value of 2.8 pA/pF in order to describe the mean current density remaining in the presence of 300 µM tolbutamide.

The decrease of the EGFP-induced fluorescence intensity in hERG/EGFP-expressing HEK 293 cells after obtaining the whole-cell configuration was calculated according to the following equation:

3 $$\frac{F}{{F_{max} }} = \left( {1 - a} \right) \times e^{{ - \frac{t - b}{\tau }}} { } + { }a$$

where F is the EGFP-induced fluorescence intensity at different times (t) after obtaining the whole-cell configuration, F_max_ is the mean value of the EGFP-induced fluorescence intensity obtained during the first three scans before rupture of the membrane patch (control), b represents the time from the beginning of the experiment (giga-seal formation) until rupture of the membrane patch for establishment of the whole-cell configuration (180 s), constant a describes the fluorescence intensity value of the cells after prolonged dialysis of the cell interior by the pipette solution, and τ designates the time constant for the decrease of the EGFP-induced fluorescence intensity. Constant a includes the autofluorescence of the cells, which was < 10% of the EGFP-induced fluorescence intensity.

The increase of the fluorescence intensity in RINm5F cells under whole-cell patch-clamp recording conditions after administration of 10 nM Bodipy-glibenclamide was calculated as follows:

4 $$\frac{F}{{F_{cont} }} = \left( {\frac{{F_{max} }}{{F_{cont} }} - 1} \right) \times \left( {1 - e^{{ - \frac{t - b}{\tau }}}} \right) + 1$$

where F is the fluorescence intensity at different times (t) after the addition of 10 nM Bodipy-glibenclamide to the bath solution, F_cont_ is the mean value of the fluorescence intensity obtained during the first three scans before application of Bodipy-glibenclamide (background fluorescence), F_max_ represents the calculated maximum asymptotic value of the Bodipy-glibenclamide-induced fluorescence intensity, b designates the time from the beginning of the experiment (establishment of the whole-cell configuration) until application of Bodipy-glibenclamide, and τ is the time constant for the Bodipy-glibenclamide-induced increase of fluorescence intensity.

The decrease of the fluorescence intensity in RINm5F cells under whole-cell patch-clamp recording conditions after washout of Bodipy-glibenclamide was calculated according to the equation:

5 $$\frac{F}{{F_{cont} }} = \left( {\frac{{F_{max} }}{{F_{cont} }} - a} \right) \times e^{{ - \frac{t - b}{\tau }}} + {\text{a}}$$

F is the fluorescence intensity at different times (t) after washout of 10 nM Bodipy-glibenclamide, F_cont_ is the mean value of the fluorescence intensity obtained during the first three scans before application of Bodipy-glibenclamide (background fluorescence), F_max_ is the maximum fluorescence intensity during application of 10 nM Bodipy-glibenclamide, b represents the time from the establishment of the whole-cell configuration until washout of Bodipy-glibenclamide, τ is the time constant for the decrease of the Bodipy-glibenclamide-induced fluorescence intensity, and constant a designates the fluorescence intensity value of the cells after prolonged dialysis of the cell interior by the pipette solution.

In FCS experiments, the intensity fluctuations were analyzed by an autocorrelation function (G(t)), as implemented in the program ConfoCor 2, using the following two-component model:

6 $$G\left( t \right) = 1 + { }\frac{{\left( {1 - TA + TA \cdot e^{{ - \frac{t}{to}}} } \right)}}{{\left( {1 - TA} \right)}} \cdot \frac{1}{N}\left( {\frac{Y}{{1 + \frac{t}{{\tau_{1} }}}} \cdot \frac{1}{{\sqrt {\left( {1 + \frac{t}{{\tau_{1} \cdot S^{2} }}} \right)} }} + \frac{1 - Y}{{1 + \frac{t}{{\tau_{2} }}}} \cdot \frac{1}{{\sqrt {\left( {1 + \frac{t}{{\tau_{2} \cdot S^{2} }}} \right)} }}} \right)$$

where TA represents the average fraction of dye molecule in the triplet state with relaxation time t_o_, N is the total average number of fluorescent molecules in the observation volume, Y and τ_1_ are the fraction and diffusion time of the fast component, 1-Y and τ_2_ designate the fraction and diffusion time of the slow component, and S is the structure parameter defining the ratio between the axial (ω_z_) and the lateral (ω_xy_) half-axes of the observation volume. Since the autofluorescence of the cells was < 10% of the EGFP-induced fluorescence intensity, no correction for background fluorescence intensity was performed.

The value of the diffusion coefficient for rhodamine 6G (D_Rho6G_ = 2.8^.^ 10^–10^ m^2.^ s^−1^) was used for determination of the parameters ω_xy_ and S prior to the experiments. The diffusion time constant is related to ω_xy_ through

7 $$\tau_{d} = \frac{{\omega_{xy}^{2} }}{4D}$$

The structure parameter S and the detection volume obtained with 50 nM rhodamine 6G were 6.3 and 0.4 fl, respectively. The structure parameter S was fixed in autocorrelation functions of further experiments. The fast (D_1_) and slow (D_2_) diffusion coefficients of the hERG/EGFP fusion protein expressed in HEK 293 cells were calculated from the measured diffusion times (τ_1_ and τ_2_) according to Eq. (7).

Values in the text and figures are presented as mean values ± S.E.M. Equations were fitted using Prism 5.0 (Graphpad Software, San Diego, CA, USA). Significances were calculated by the two-tailed non-paired t test for single comparisons and by ANOVA with Bonferroni correction for multiple comparisons. P < 0.05 was considered significant.

### Determination of the Terfenadine Concentrations in Test Solutions Using HPLC

The isocratic method for the detection and quantification of terfenadine in extracellular and intracellular solutions was established by using the chromatographic conditions described in the European Pharmacopoeia 8.0 (2008). Chromatographic analysis was achieved using an Agilent 1100 series HPLC consisting of vacuum degasser G1322A, quaternary pump G1311A, auto-sampler G1329A, column oven G1316A, and diode array detector G1315A. The system was controlled by Chemstation software Version B.04.03. Reverse phase chromatography was performed at room temperature (20–22 °C) using an Agilent Zorbax SB-C8, 5 µm, 3.0 mm^.^ 250 mm column. The column was protected with a guard cartridge (SecurityGuard™) filled with a C8 Octyl MOS (Phenomenex). The mobile phase was delivered isocratically at a flow rate of 0.4 ml/min. The mobile phase contained acetonitrile and diethylammonium phosphate buffer. The diode array detector G1315A was set at 217 nm. The auto-sampler cooling system was programmed at room temperature (20—22 °C) and injection was adjusted to 100 µl for extracellular and 40 µl for intracellular samples.

### Drugs and Solutions

The bath solution (A) contained (in mM): 140 NaCl, 5.6 KCl, 1.2 MgCl_2_, 2.6 CaCl_2_, and 10 HEPES titrated to pH = 7.40 with NaOH. The pipette (solution B) contained (in mM): 140 KCl, 1 MgCl_2_, 2 CaCl_2_, 10 EGTA, and 5 HEPES titrated to pH = 7.15 with KOH (free [Ca^2+^] = 50 nM; free [Mg^2+^] = 0.7 mM). Solution (B) to which 0.3 mM ATP was added was the pipette solution (C) for whole-cell experiments on RINm5F cells, and solution (B) to which 5 mM ATP and 4 mM MgCl_2_ were added was the pipette solution (D) for whole-cell experiments on hERG channels. Low-binding pipette tips (Sorenson Bioscience, Salt Lake City, UT, USA) were used for filling the glass patch pipettes with solutions containing the test substances.

Stock solutions of 30 mM terfenadine (Sigma, St. Louis, MO, USA) and 3 mM dofetilide (Sequoia, Pangbourne, UK) were prepared in DMSO. Stock solutions of 30 mM tolbutamide were prepared daily in 50 mM NaOH or KOH and aliquots were added to solutions A or C, respectively, to give the final concentrations, and the pH values were readjusted. Bodipy-glibenclamide was purchased from Molecular Probes and a stock solution of 100 µM Bodipy-glibenclamide was prepared in DMSO and applied to solution (A) to give the final concentration. Cyclosporine A (Sigma) was prepared as a 10 mM stock solution in DMSO and diluted in solution (A) to a final test concentration of 10 µM. Chemicals used for the chromatography were acetonitrile as HPLC grade (Sigma), phosphoric acid 85% (Merck, Darmstadt, Germany), and diethylamine (Roth, Karlsruhe, Germany).

## Results

### Effects of Terfenadine and Dofetilide on hERG Channels After Application via the Pipette Solution

**Fig. 1:**
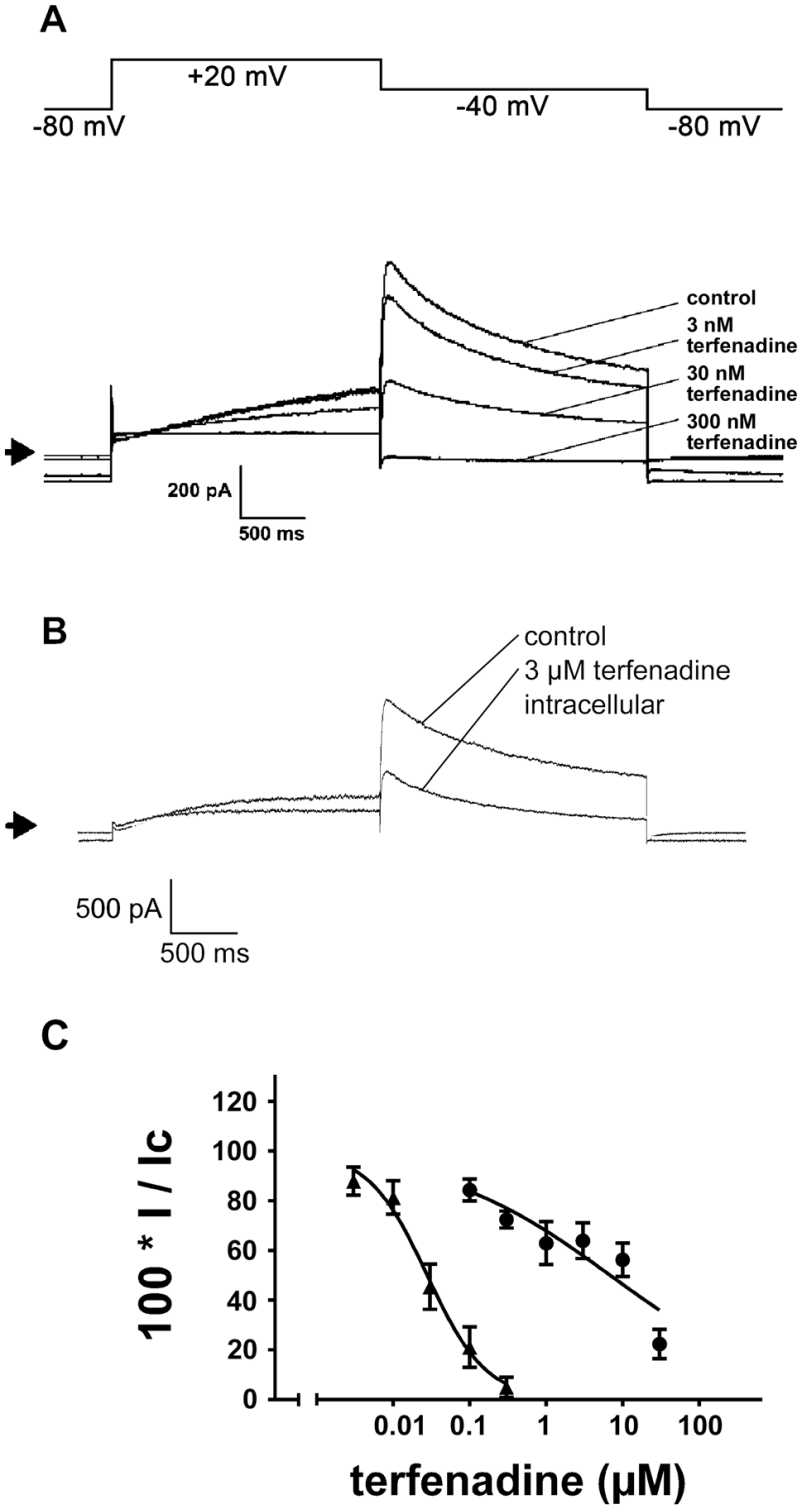
Effects of application of terfenadine via either the bath or pipette solution on hERG currents using the whole-cell configuration of the patch-clamp technique. **A** the voltage-clamp protocol used to study hERG currents (upper trace). The lower traces show the effects of terfenadine applied via the bath solution on hERG currents. The arrow indicates the zero current level. The extent of block of hERG peak tail currents at terfenadine concentrations of 3, 30, and 300 nM was 17.7, 57.3, and 94.9%, respectively. **B** hERG current traces at times 0 min (control) and 10 min after breaking into the cell in the presence of 3 µM terfenadine in the pipette solution; the extent of hERG peak tail current block was 47.1%. **C** concentration–response relationships for the inhibition of hERG peak tail currents by terfenadine applied via either the bath or pipette solution. The ordinate represents current amplitudes in the presence of different concentrations of terfenadine applied via either the bath or pipette solution (I) in percent of the current amplitudes obtained either before extracellular application of terfenadine or at time 0 min after breaking into the cells with intracellular application of terfenadine (control; I_c_). The abscissa indicates the concentrations of terfenadine (logarithmic scale). Symbols represent means and the vertical lines the S.E.M. for terfenadine applied either via the bath (▲) or via the pipette (●) solution. Numbers of observations were between 6 and 9 at each concentration tested. The lines are fits to Eq. (1)

Terfenadine administered via the bath solution inhibited hERG peak tail currents concentration-dependently with values calculated for IC_50_ of 27.7 nM (95% confidence interval: 24.4–31.3) and for the Hill coefficient (n) of 1.1 (95% confidence interval: 1.0–1.3; Fig. 1, Friemel and Zünkler 2010). In contrast, the effects of terfenadine applied via the pipette solution on hERG peak tail currents were much weaker, and when determined 10 min after breaking into the cells the IC_50_ value was 6.9 µM (95% confidence interval: 3.3–14.2 µM) and the Hill coefficient was 0.39 (95% confidence interval: 0.24–0.53 (Fig. 1)). For dofetilide applied via the pipette solution, values for IC_50_ of 55 nM (95% confidence interval: 46 – 66 nM) and for the Hill coefficient of 1.0 (95% confidence interval: 0.8–1.1) were obtained (results not shown). By comparison, after application via the bath solution, an IC_50_ value of 12.9 nM has been determined for dofetilide-induced block of hERG peak tail currents (Friemel and Zünkler 2010).

### Effects of Extra- and Intracellularly Applied Terfenadine on K_ATP_ Channels from RINm5F Cells

**Fig. 2:**
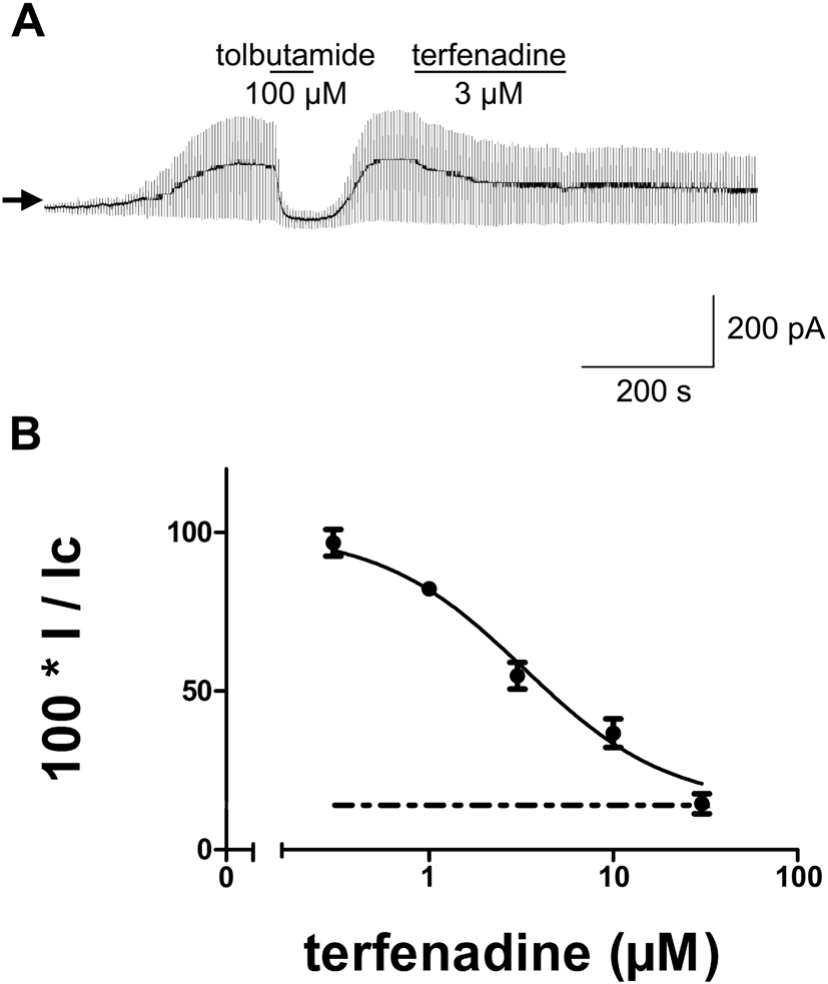
Effects of terfenadine applied via the bath solution on K_ATP_ currents from RINm5F cells using the whole-cell configuration of the patch-clamp technique. The holding potential was –70 mV and current responses to ± 10 mV pulses are shown. **A** in the example shown, current amplitudes increased during the first minutes of recording due to the washout of ATP from the cytoplasm. Tolbutamide (100 µM) was added to the bath solution during the period indicated by the first horizontal bar and reversibly blocked current amplitudes by 89.1%. Terfenadine (3 µM) was added to the bath solution during the period indicated by the second horizontal bar and blocked current amplitudes by 38.9%. After washout of terfenadine, channel activity did not recover. **B** concentration–response relationship for the inhibition of whole-cell K_ATP_ currents from RINm5F cells by terfenadine applied via the bath solution. The ordinate represents current amplitudes in the presence of different concentrations of terfenadine applied via the bath solution (I) in percent of the current amplitudes in the absence of terfenadine (control; I_c_). The abscissa indicates the concentrations of terfenadine (logarithmic scale). Points represent means and the vertical lines the S.E.M. Numbers of observations were between 4 and 10 at each concentration tested. The line is a fit to Eq. (2). The broken line indicates the mean value of the current amplitudes in the presence of 100 µM tolbutamide in percent of control current amplitudes (14.1%)

Terfenadine applied via the bath solution concentration-dependently inhibited K_ATP_ currents from RINm5F cells with an IC_50_ value of 3.3 µM (95% confidence interval: 2.6–4.2 µM) and a slope parameter (n) of 1.1 (95% confidence interval: 0.8–1.4; Fig. 2). After washout of terfenadine, recovery of channel activity was slow (Fig. 2A). Therefore, no correction for the run-down of channel activity was possible.

**Fig. 3:**
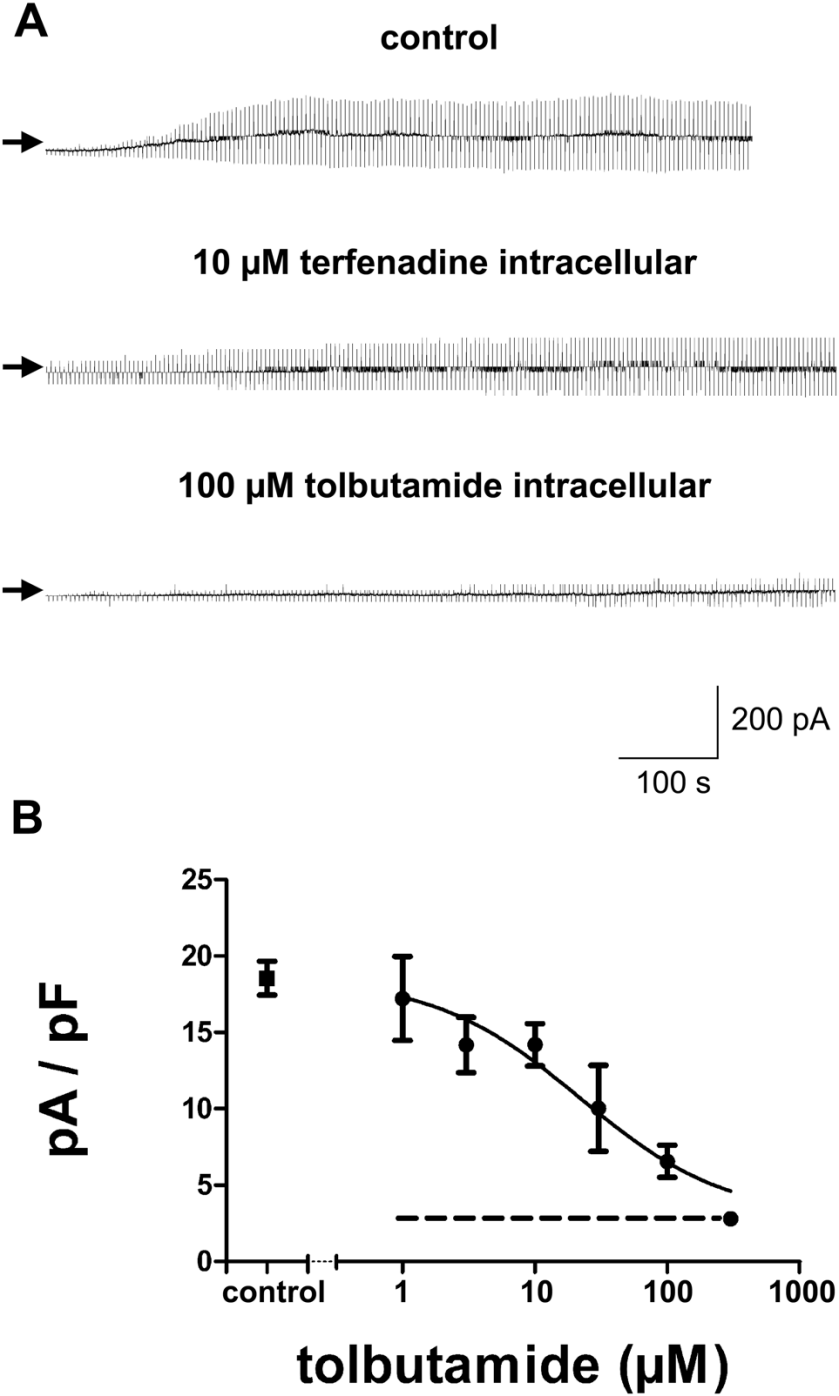
Effects of terfenadine and tolbutamide applied via the pipette solution on K_ATP_ currents from RINm5F cells using the whole-cell configuration of the patch-clamp technique. The holding potential was –70 mV and current responses to ± 10 mV pulses are shown. **A** time courses of K_ATP_ currents during 12–13 min after breaking into the cell in the absence (upper trace) and the presence of 10 µM terfenadine (middle trace) or 100 µM tolbutamide (lower trace) in the pipette solution. Maximum currents in response to 10 mV depolarizing pulses were divided by the cell capacitance and were 19.8 pA/pF under control conditions, 16.8 pA/pF in the presence of 10 µM terfenadine in the pipette solution and 6.5 pA/pF in the presence of 100 µM tolbutamide in the pipette solution. **B** concentration–response relationship for the inhibition of whole-cell K_ATP_ currents from RINm5F cells by tolbutamide applied via the pipette solution. The ordinate represents current densities in the absence of test substances in the pipette solution (control, I_c_; mean value 18.6 pA/pF) and in the presence of different concentrations of tolbutamide applied via the pipette solution (I). The abscissa indicates the concentrations of tolbutamide (logarithmic scale). Points represent means and the vertical lines the S.E.M. Numbers of observations were between 5 and 9 at each concentration tested. The line is a fit to Eq. (2)

Figure 3 shows the effects of terfenadine or tolbutamide applied via the pipette solution on K_ATP_ currents from RINm5F cells. Maximum current density in response to 10 mV depolarizing pulses obtained within 10–15 min after breaking into the cells was 18.6 ± 1.1 pA/pF (n = 17) under control conditions and 17.7 ± 2.1 pA/pF (n = 8) in the presence of 10 µM terfenadine in the pipette solution (corresponding to 5.7% block, taking the leak currents of 2.8 pA/pF into account). The application of tolbutamide via the pipette solution concentration-dependently blocked K_ATP_ currents from RINm5F cells with an IC_50_ value of 22.0 µM (95% confidence interval: 11.3 – 42.7 µM) and a slope parameter (n) of 0.8 (95% confidence interval: 0.4 – 1.2).

### Effects of P-glycoprotein Inhibition on the Effects of Intracellularly Applied Terfenadine on hERG Channels

**Fig. 4:**
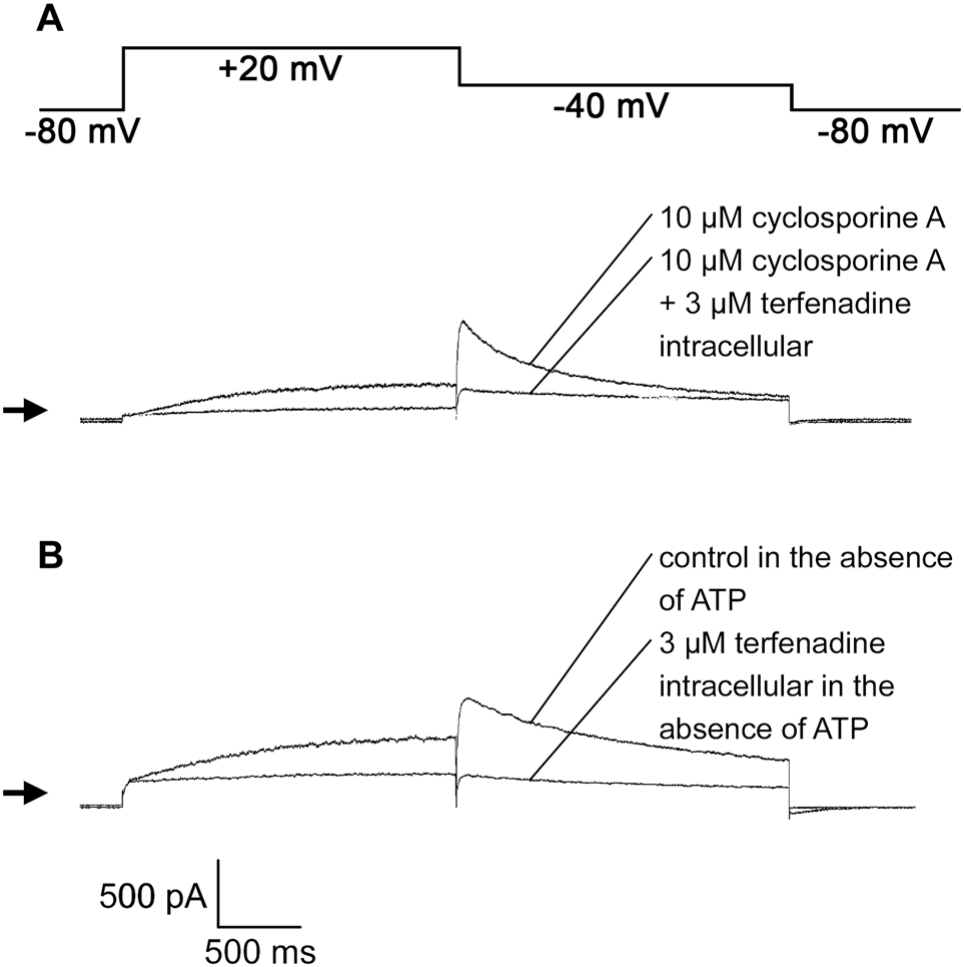
Effects of terfenadine applied via the pipette solution for 10 min on hERG currents in the presence of the P-glycoprotein inhibitor cyclosporine A in the bath solution and in the absence of MgATP in the pipette solution. **A** the voltage-clamp protocol used to study hERG currents (upper trace). The lower traces show the effects of 3 µM terfenadine applied via the pipette solution in the presence of 10 µM cyclosporine A in the bath solution on hERG currents. The extent of block induced by 3 µM terfenadine applied via the pipette solution was 77.3% in the presence of 10 µM cyclosporine A. **B** hERG current traces at times 0 min (control) and 10 min after breaking into the cell in the presence of 3 µM terfenadine in the pipette solution in the absence of MgATP. The extent of block induced by 3 µM terfenadine was 76.4% in the absence of MgATP in the pipette solution (**B**)

In order to study whether P-glycoprotein-mediated efflux of terfenadine is responsible for the low potency of intracellularly applied terfenadine to block hERG channels, the activity of P-glycoprotein in the plasma membrane was inhibited either by the presence of cyclosporine A in the bath solution or by the absence of MgATP in the pipette solution. 10 µM cyclosporine A applied via the bath solution inhibited hERG peak tail currents by 44.1 ± 6.5% (n = 7). 10 min after breaking into the cell, 3 µM terfenadine applied via the pipette solution inhibited hERG peak tail currents by 80.5 ± 1.9% (n = 5) in the presence of 10 µM cyclosporine A (Fig. 4A) and by 72.8 ± 6.5% (n = 10) when MgATP was absent in the pipette solution (Fig. 4B). These values were significantly different (P < 0.05) from the extent of block induced by 3 µM terfenadine obtained in the presence of MgATP in the pipette solution (36.1 ± 7.2%, n = 8; Fig. 1).

### Diffusion of hERG/EGFP Fusion Proteins Into the Pipette Solution Under Whole-Cell Patch-Clamp Conditions

**Fig. 5:**
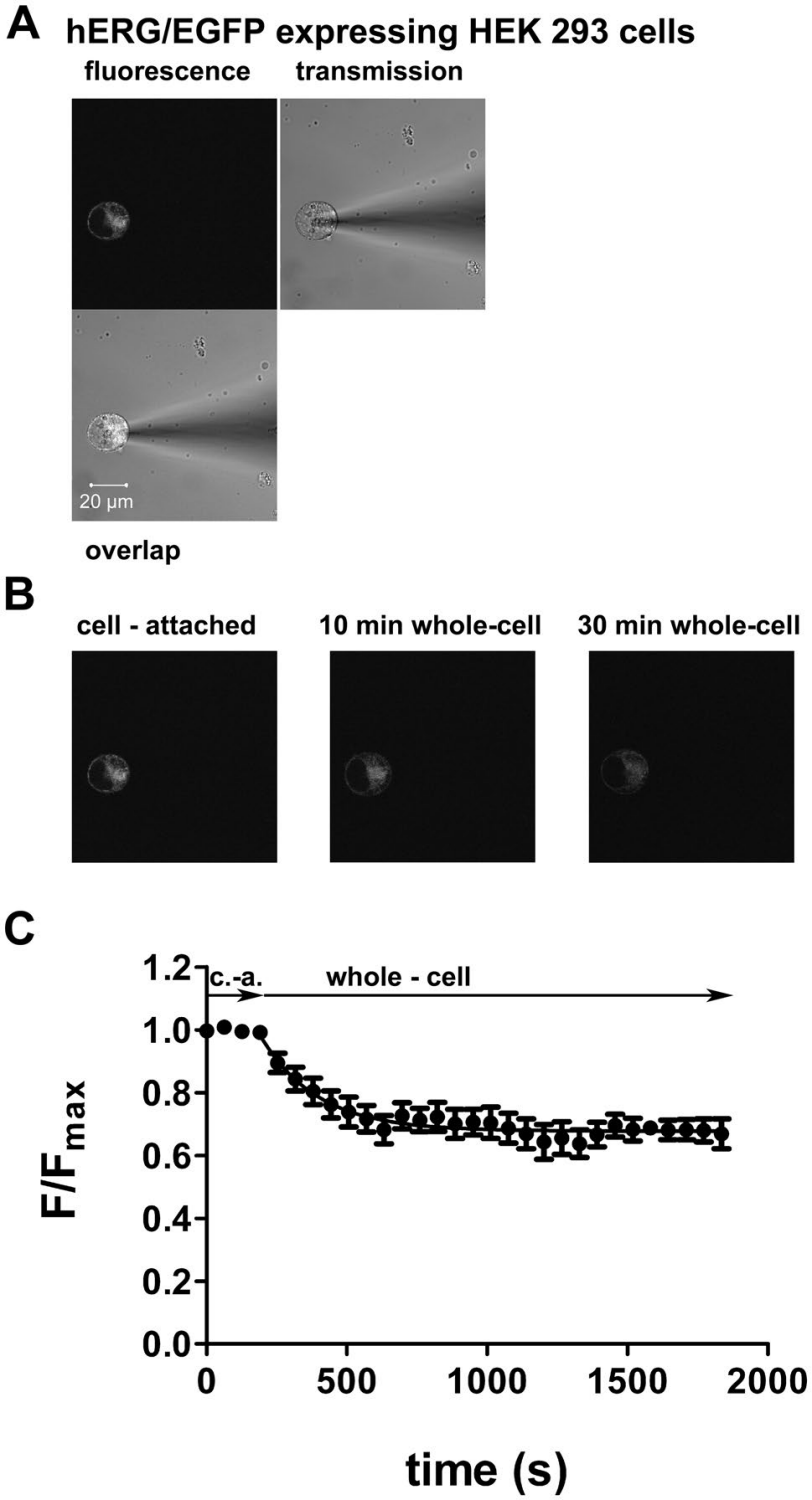
Decrease of the EGFP-induced fluorescence intensity in hERG/EGFP fusion protein-expressing HEK 293 cells after obtaining the whole-cell configuration evaluated using a laser-scanning microscope. **A** EGFP-induced fluorescence intensity in a hERG/EGFP fusion protein-expressing HEK 293 cell (upper left), transmission image of the same cell with an attached patch pipette (upper right), and fusion image of both (lower left). **B** time course of the EGFP-induced fluorescence intensity in hERG/EGFP fusion protein-expressing HEK 293 cells in the cell-attached configuration (left) and 10 min (middle) or 30 min (right), respectively, after membrane rupture. Fluorescence intensity values determined from a region of interest (ROI) located over the entire cell were 32 (left), 27 (middle), and 20 (right). **C** mean values ± S.E.M. of EGFP-induced fluorescence intensity in hERG/EGFP fusion protein-expressing HEK 293 cells after obtaining the whole-cell configuration as a function of time obtained from 11 experiments. Ordinate: EGFP-induced fluorescence intensity normalized to the mean value of the three time points before rupture of the membrane patch (F/F_max_; c.-a. cell attached); abscissa: time. Points represent means and the vertical lines the S.E.M. The time-dependent decrease of the EGFP-induced fluorescence intensity after membrane rupture was fitted to a single-exponential function (Eq. 3)

To study whether intracellularly located hERG channels are available for the binding of terfenadine applied via the pipette solution in whole-cell experiments, the diffusion of hERG/EGFP fusion proteins expressed in HEK 293 cells into the pipette solution was investigated under whole-cell conditions (Fig. 5). Fitting a mono-exponential function (Eq. 3) to the decrease of the EGFP-induced fluorescence intensity yielded values for τ of 201 s (95% confidence interval: 151–301 s) and for constant a (plateau) of 0.68 (95% confidence interval: 0.65–0.71; Fig. 5C). Therefore, only 32% of the hERG/EGFP fusion proteins are freely diffusible in HEK 293 cells and available to dialysis by the patch-pipette solution.

### Diffusion of hERG/EGFP Fusion Proteins Expressed in HEK 293 Cells as Determined by FCS

**Fig. 6:**
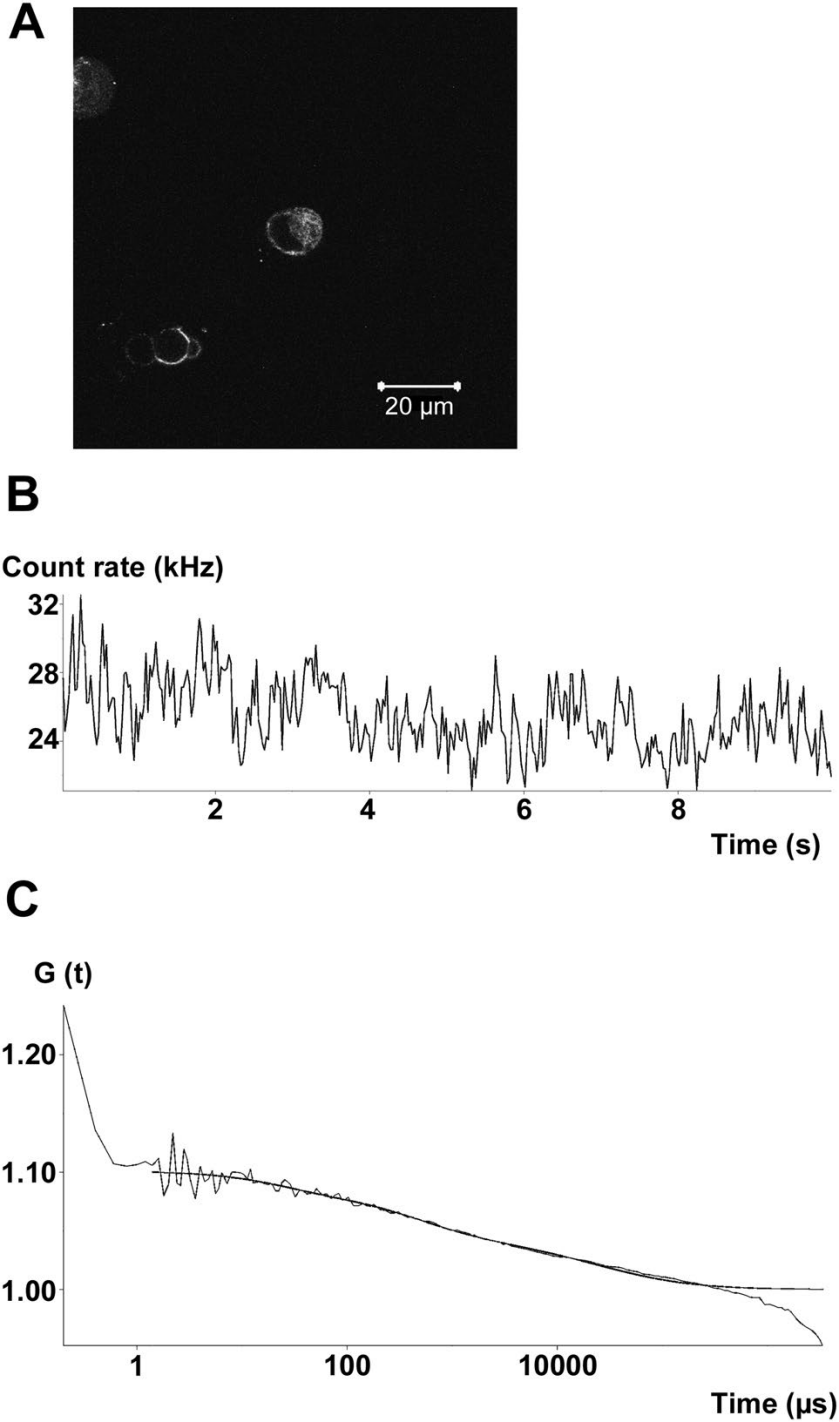
FCS experiments of hERG/EGFP channels transiently expressed in HEK 293 cells. **A** confocal image of fluorescence intensity from HEK 293 cells expressing hERG/EGFP channels. **B** fluorescence intensity fluctuations (count rate) obtained from the cytosol of the cell in the middle of the image. **C** fluorescence autocorrelation function (G(t)) of hERG/EGFP channels expressed in HEK 293 cells fitted with a two-component model. hERG/EGFP channels had a count rate of 25.5 kHz/molecule, and the diffusion time constants in the cytosol of this example were 0.46 and 29.4 ms, respectively. In addition, a triplet state can be seen at short correlation times with a lifetime of 24 µs

The diffusion of hERG/EGFP channels expressed in HEK 293 cells was determined using FCS. In 17 cells obtained from three transfections, two components for the motion of hERG/EGFP molecules in the cytosol of HEK 293 cells were observed (Fig. 6): a fast component with a diffusion time (τ_1_) of 0.31 ± 0.03 ms and a slow component with a diffusion time (τ_2_) of 18.1 ± 3.7 ms, corresponding to diffusion coefficients of 39 ± 4^.^ 10^–12^ m^2.^ s^−1^ for D_1_ and 67 ± 14^.^ 10^–14^ m^2.^ s^−1^ for D_2_, respectively. The proportion of the D_1_ and D_2_ components was 63.1 ± 3.9% and 36.8 ± 3.9%, respectively. The count rate per fluorescent molecule was 1.6 ± 0.2 kHz, and the number of hERG/EGFP molecules in the detection volume was 41.1 ± 11.5, corresponding to a concentration of 184 ± 52 nM.

### Binding of Extracellularly Applied Bodipy-Glibenclamide to RINm5F Cells Under Whole-Cell Patch-Clamp Conditions

**Fig. 7:**
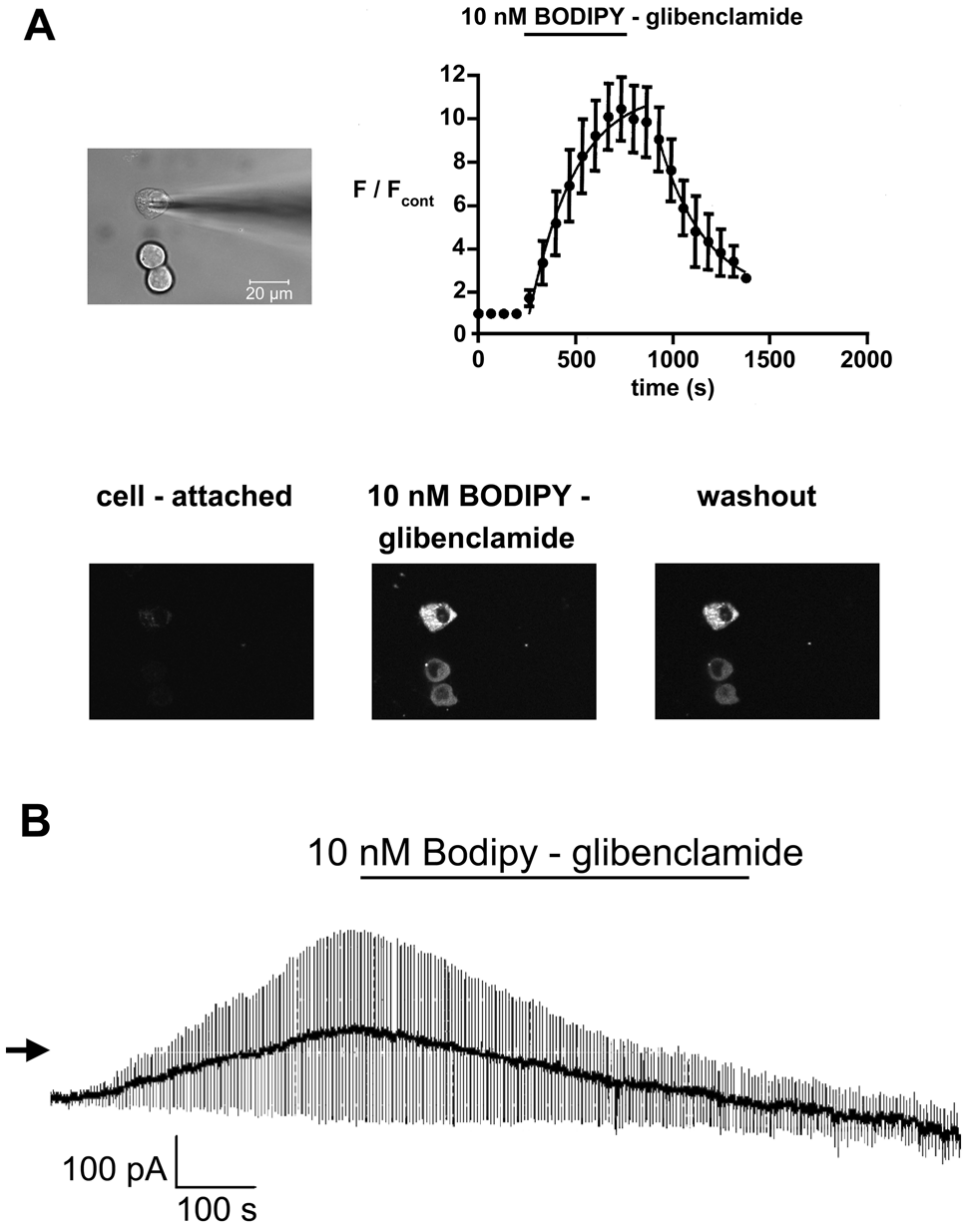
Increase of the Bodipy-glibenclamide-induced fluorescence intensity in RINm5F cells in the whole-cell configuration evaluated using a laser-scanning microscope. **A** upper left: Transmission image of RINm5F cells with an attached recording pipette in the whole-cell configuration. Lower: Confocal images of the fluorescence of the cells before (left) and 6 min after administration of 10 nM Bodipy-glibenclamide (middle) and after 5 min washout of Bodipy-glibenclamide (right). Fluorescence intensity values determined from a region of interest (ROI) located over the entire cell were 10 before, 97 after 6 min administration of Bodipy-glibenclamide and 73 after 5 min of washout of Bodipy-glibenclamide. Upper right: Mean values ± S.E.M. of fluorescence intensity from 7 RINm5F cells in the whole-cell configuration as a function of time before, during administration of 10 nM Bodipy-glibenclamide and after washout. 10 nM Bodipy-glibenclamide was present throughout the period indicated by the horizontal bar. Ordinate: fluorescence intensity normalized to the mean value of the three time points before administration of 10 nM Bodipy-glibenclamide (F/F_cont_); abscissa: time. Points represent means and the vertical lines the S.E.M. The time-dependent increase of the Bodipy-glibenclamide-induced fluorescence intensity was fitted to Eq. (4), and the time-dependent decrease of the fluorescence intensity after washout of Bodipy-glibenclamide was fitted to Eq. (5). **B** effects of 10 nM Bodipy-glibenclamide on K_ATP_ currents from RINm5F cells using the whole-cell configuration determined simultaneously with the laser-scanning microscopy. The holding potential was –70 mV and current responses to ± 10 mV pulses are shown. The extent of block induced by 10 nM Bodipy-glibenclamide was 63% in this example, and the extent of block was still 75% after 5 min washout of Bodipy-glibenclamide

In order to further study whether intracellular components are dialyzed by the pipette solution under whole-cell conditions, the binding of Bodipy-glibenclamide applied via the bath solution to RINm5F cells was investigated in 7 experiments under whole-cell patch-clamp conditions (Fig. 7). The time-dependent increase of the Bodipy-glibenclamide-induced fluorescence intensity was fitted to Eq. (4): the value for constant b was fixed to 261 s, the value calculated for τ was 225 s (95% confidence interval: 126–1018 s), and the value calculated for F_max_ was 11.5 (95% confidence interval: 8.1–14.9). Similar values were obtained in 5 intact RINm5F cells exposed to Bodipy-glibenclamide: the value calculated for τ was 315 s (95% confidence interval: 196–810 s), and the value calculated for F_max_ was 10.7 (95% confidence interval: 7.6–13.9; results not shown). After washout of Bodipy-glibenclamide, the time-dependent decrease of the Bodipy-glibenclamide-induced fluorescence intensity from RINm5F cells under whole-cell conditions was fitted to Eq. (5): the value for constant b was fixed to 915 s, the value for F_max_ was fixed to 9.2, the value calculated for τ was 245.2 s, and the value calculated for constant a was 2.0 (Fig. 7A). In contrast to the decrease of the fluorescence intensity after washout of Bodipy-glibenclamide, block of K_ATP_ currents by 10 nM Bodipy-glibenclamide was nearly complete and not reversible after washout of Bodipy-glibenclamide (Fig. 7B). The extent of block of K_ATP_ currents induced by 10 nM Bodipy-glibenclamide was 53 ± 5% 6 min after application of 10 nM Bodipy-glibenclamide and further increased to 70 ± 6% after 5 min washout, which might be explained by the run-down of channel activity.

### Effects of Extra- and Intracellularly Applied Terfenadine on hERG (Y652A) Channels

**Fig. 8:**
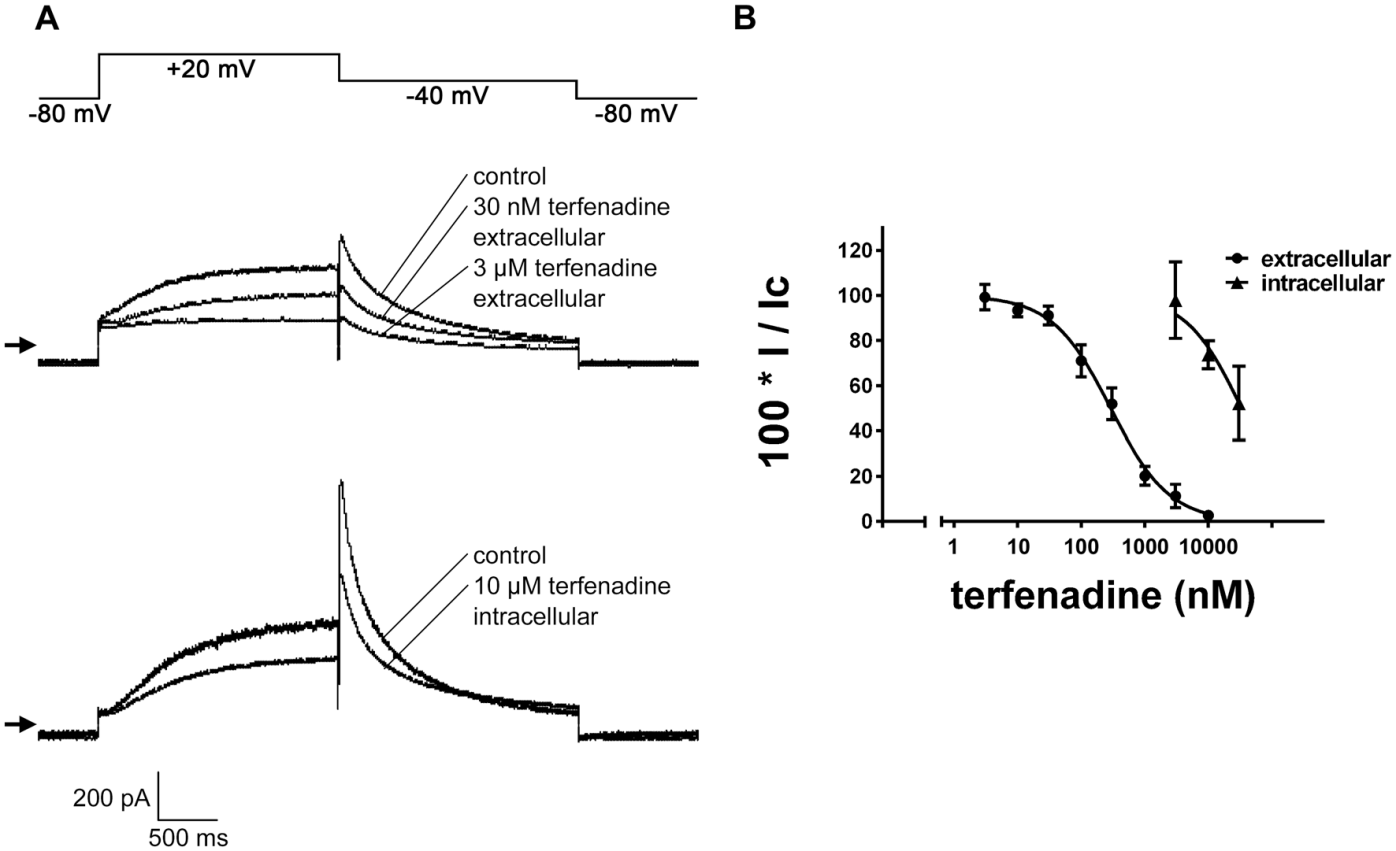
Effects of terfenadine administered either via the bath solution or via the pipette solution on hERG (Y652A) currents using the whole-cell configuration of the patch-clamp technique. **A** the voltage-clamp protocol used to study hERG (Y652A) currents (upper trace). Effects of terfenadine administered via the bath solution on hERG (Y652A) currents (middle traces). The extent of block was 39.6% at 30 nM and 86.0% at 3 µM terfenadine, respectively, in this example. Representative current traces at times 0 min (control) and 10 min after breaking into the cell in the presence of 10 µM terfenadine in the pipette solution (lower traces). The extent of block was 33.8% in this example. **B** concentration–response relationships for the inhibition of hERG (Y652A) peak tail currents by terfenadine administered either via the bath solution (●) or via the pipette solution for 10 min (▲). The ordinate represents current amplitudes in the presence of different concentrations of terfenadine (I) in percent of the current amplitudes either determined before extracellular administration of terfenadine or directly after breaking into the cells for intracellular administration of terfenadine (control; I_c_). The abscissa indicates the concentrations of terfenadine (logarithmic scale). Symbols represent means and the vertical lines the S.E.M.. Numbers of observations were between 5 and 7 after extracellular application and between 3 and 5 after intracellular application at each concentration tested. The lines are fits to Eq. (1)

Since Y652 is an important binding site for terfenadine on hERG channels (Mitcheson et al. 2000), we tested the effects of extra- and intracellularly applied terfenadine on mutated hERG (Y652A) channels in order to determine whether binding of terfenadine to the canonical binding site on hERG channels located intracellularly reduces the concentration of terfenadine beneath the plasma membrane. The concentration–response relationship for the inhibition of hERG (Y652A) peak tail current amplitudes by terfenadine administered via the bath solution fitted according to Eq. (1) yielded values for IC_50_ of 291 nM (95% confidence interval: 221–382 nM) and for the Hill coefficient (n) of 0.9 (95% confidence interval: 0.7–1.2; Fig. 8). Terfenadine administered via the pipette solution for 10 min inhibited hERG (Y652A) peak tail currents with an extrapolated IC_50_ value of 33 µM (95% confidence interval: 12–92 µM) at a fixed Hill coefficient of 1 (Fig. 8).

## Discussion

The present study demonstrates that terfenadine applied via the pipette solution in the whole-cell configuration is blocking both hERG and K_ATP_ currents with a much lower potency than when applied via the bath solution (Figs. 1–3, Tables 1 and 2). This observation is surprising since the binding sites for terfenadine on hERG channels (Mitcheson et al. 2000) and on K_ATP_ channels (Nishio et al. 1998) are accessible from the cytoplasmic side of the plasma membrane, and terfenadine (pKa value of 9.86) is predominantly (> 99%) charged at the pH value of the pipette solution (7.15) used in our whole-cell experiments.

**Table 1:** IC_50_ values for the inhibition of hERG peak tail currents by terfenadine and dofetilide obtained after application either via the bath solution or via the pipette solution in whole-cell patch-clamp experiments on hERG channel expressing HEK 293 cells

	Whole-cell, bath solution	Whole-cell, pipette solution
terfenadine	27.7 nM^a^	6.9 µM^b^
dofetilide	12.9 nM^a^	55 nM

^a^Fig.1,Friemel and Zünkler (2010)

^b^Fig. 1

**Table 2:** IC_50_ values for the inhibition of pancreatic ß-cell K_ATP_ currents by terfenadine and tolbutamide obtained after application either via the bath solution or via the pipette solution in whole-cell experiments or after application via the bath solution in inside-out patch-clamp experiments

	Whole-cell, bath solution	Whole-cell, pipette solution	Inside-out, bath solution
terfenadine	3.3 µM^a^	** > > **10 µM^b^	1.2 µM^c^
tolbutamide	7 µM^d^	22 µM^e^	4.2 µM^f^

^a^Fig. 2

^b^Fig. 3

^c^Zünkler et al. (2000); RINm5F cells

^d^Trube et al. (1986); mouse pancreatic ß-cells

^e^Fig. 3

^f^Zünkler et al. (1988); mouse pancreatic ß-cells, in the presence of 1 mM MgADP

Terfenadine adsorbs to the materials used in perfusion systems (Bridgland-Taylor et al. 2006; Lu et al. 2012; Goineau et al. 2013; Orvos et al. 2019; Brinkwirth et al. 2020). This property might be caused by its high lipophilicity (logP (octanol/water partition coefficient for the non-ionized form) value of 5.8), since substances with logP values > 5 may stick to the equipment (Qu et al. 2011). The variable IC_50_ values (7–204 nM) reported for terfenadine-mediated block of hERG currents (Polak et al. 2008) might be explained by non-specific binding to the different materials used in perfusion systems. Therefore, in our experiments, care was taken to avoid adsorption of terfenadine to the laboratory equipment: stock solutions were prepared in glass containers, dilutions of terfenadine were performed using coated plastic tips, bath application of terfenadine was performed at a constant perfusion of 2 ml/min using PVC tubes, and patch pipettes were filled using low binding pipette tips. The concentrations of terfenadine determined in both the bath and patch-pipette solutions by HPLC were at least 80% of the nominal terfenadine concentrations.

In contrast to terfenadine, specific blockers of hERG channels (methane sulfonamide class III antiarrhythmic substances E-4031 (Zhang et al. 2010; Sale et al. 2017) and dofetilide (Du et al. 2011; present study)) and K_ATP_ channels (tolbutamide; Fig. 3 and Table 2) inhibit the currents with similar potencies from both sides of the plasma membrane.

Pusch and Neher (1988) determined the rates of diffusional exchange between a patch pipette and small cells in the whole-cell configuration. Using an empirical equation developed by the authors, the values for the diffusion times τ are calculated as 41 s for terfenadine and 40 s for dofetilide at a mean pipette access resistance of 8.8 MΩ in the present whole-cell experiments on hERG channels. Therefore, after application via the pipette solution, the concentrations of these substances in the cell interior might reach a steady state within a few minutes after rupture of the membrane patch.

One possible explanation for the low potency of terfenadine applied via the pipette solution regarding block of hERG and K_ATP_ currents might be transporter-mediated efflux from HEK 293 cells. Drug efflux mediated via P-glycoprotein modulates the inhibition of hERG currents by ibutilide (McBride et al. 2009), dofetilide (Hreiche et al. 2009), and rosuvastatin (Plante et al. 2012). Terfenadine is a substrate of P-glycoprotein (Obradovic et al. 2006). P-glycoprotein is expressed in HEK 293 cells (Gow et al. 2008) which were used in the present study for the expression of hERG channels. Both in the presence of cyclosporine A in the bath solution and in the absence of MgATP from the pipette solution, the potency of 3 µM terfenadine to block hERG currents was only slightly increased (by a factor of about 2) after application via the pipette solution (Fig. 4). This indicates that P-glycoprotein-mediated efflux of terfenadine is only responsible to a minor extent for the reduced ability of intracellularly applied terfenadine to block hERG channels.

The most plausible explanation for the low hERG and K_ATP_ current-blocking potency of terfenadine applied via the pipette solution might be binding to intracellular components not dialyzable by the pipette solution in whole-cell experiments. This may prevent most terfenadine molecules from reaching their binding sites on the channels from the cytosolic side of the plasma membrane. This hypothesis was studied in experiments investigating the dialysis of intracellular components in the whole-cell configuration.

First, only 32% of the hERG/EGFP fusion proteins are dialyzable by the patch-pipette solution (Fig. 5). Second, in order to study the diffusion of fluorescently labeled ion channels in intact cells, FCS was performed on hERG/EGFP-expressing HEK 293 cells (Fig. 6). We confirmed the observation by Hayakawa et al. (2011) that there are two separate protein populations with distinct diffusion coefficients differing by a factor of about 100 in the cytosol. Hayakawa et al. suggested that the fast component represents the free form of protein, whereas the slow component represents the diffusion of proteins associated with intracellular organelles. The FCS results confirm the results of the laser-scanning microscopy experiments performed under whole-cell recording conditions (Fig. 5) that there are at least two components of hERG channels with distinct diffusion coefficients in the cytosol of HEK 293 cells. Third, Bodipy-glibenclamide applied via the bath solution induced a diffuse fluorescence both in intact RINm5F cells (Zünkler et al. 2004) and in RINm5F cells in the whole-cell configuration (Fig. 7). The observation that after washout of Bodipy-glibenclamide the fluorescence intensity decreased (Fig. 7A) whereas block of K_ATP_ currents did not recover (Fig. 7B) confirms our previous suggestion (Zünkler et al. 2004) that the major part of the Bodipy-glibenclamide-induced fluorescence intensity in RINm5F cells arises due to non-specific binding caused by its high lipophilicity (the logP value of glibenclamide is 3.1 (Panten et al. 1988)).

For both wild-type hERG and hERG (Y652A) channels, the potency of intracellularly applied terfenadine was similarly reduced by a factor of 100–200 when compared to its application via the bath solution (Figs. 1 and 8 and Table 1). This indicates that binding of terfenadine to the canonical binding site on hERG channels located intracellularly does not markedly reduce the concentration of terfenadine beneath the plasma membrane.

The most likely explanation for the reduced hERG and K_ATP_ current-blocking potency of terfenadine applied via the pipette solution is that due to its high lipophilicity, terfenadine accumulates in intracellular components which are not completely dialyzable by the pipette solution. This leads to a reduction of its concentration beneath the plasma membrane. This explanation is supported by the much higher lipophilicity of terfenadine when compared to the other substances tested in the present study: the values for pK_a_ and logP are 5.3 and 2.5 for tolbutamide (Panten et al. 1988), 7 and 9.2 (pK_a_) and 1.58 for dofetilide (Chassaing et al. 2005), and 9.86 and 5.8 for terfenadine (Dagenais et al. 2009). In the hearts of rabbits, guinea pigs and dogs, terfenadine accumulates by a factor of about 30–250 as compared to the perfusion solution (Lu et al. 2012; Moss 1999; Cavero et al. 1999; Katagi et al. 2016). Hondeghem et al. (2011) found that in isolated rabbit hearts, the application of terfenadine leads to slowly developing prolongation of the action potential duration (APD) which was explained by the slow-time course of tissue accumulation of terfenadine in the heart. Yu et al. (2015) observed that, among 15 prototypical hERG current blockers tested, terfenadine has the highest affinity to membrane phospholipids. Katagi et al. (2016) found that higher potency of hERG channel blockers goes along with increased accumulation in the myocardium. The observations of the present study add to these suggestions by demonstrating that terfenadine accumulates in intracellular components probably due to its high lipophilicity.

In conclusion, the results of this study demonstrate that terfenadine applied via the pipette solution in whole-cell patch-clamp experiments is blocking both hERG and K_ATP_ currents at a much lower potency than when applied via the bath solution. P-glycoprotein-mediated efflux of terfenadine plays only a minor role in this phenomenon. Two observations indicate that intracellular components are not completely dialyzable by the recording patch pipette: First, 68% of the hERG/EGFP fusion proteins expressed in HEK 293 cells are not or only slowly dialyzable by the pipette solution and second, Bodipy-glibenclamide applied via the bath solution induces an increase in fluorescence intensity in RINm5F cells under whole-cell conditions. The results of the electrophysiological experiments using mutated hERG (Y652A) channels indicate that the binding of terfenadine to intracellular components is probably due to its high lipophilicity and not caused by its binding within the pore of hERG channels located intracellularly. We suggest that substances with high lipophilicity are not freely diffusible inside the cell, but there might exist steep concentration gradients within the cell and in the sub-membrane space. We propose that the effects of highly lipophilic ion channel blockers which reach their binding sites from the cytosolic side of the plasma membrane are slowly reversible partly due to an accumulation in intracellular components. The effects of lipophilic hERG current blockers like terfenadine on the ECG (QT interval prolongation) in humans might, therefore, not coincide with their maximum plasma concentrations, but might be delayed due to accumulation in intracellular compartments. This behavior has recently been shown by Antzelevitch’s group demonstrating that for oliceridine (a biased ligand at the µ-opioid receptor) an intracellular accumulation in cardiac myocytes leads to an increasing hERG current block and to delayed effects on cardiac repolarization, i.e., hysteresis (Burashnikov et al. 2021).

## Data Availability

The datasets used and/or analyzed during the current study are available from the corresponding author on reasonable request.
